# Clinical characteristics of varicella-zoster virus central nervous system infection in 108 unimmunocompromised patients

**DOI:** 10.3389/fneur.2025.1554954

**Published:** 2025-02-24

**Authors:** Xiaojiao Ci, Jihong Zhang, Jie Lu, Xinyang Qi, Yifang Ma, Weiguo Liu, Jingping Shi

**Affiliations:** Department of Neurology, Affiliated Nanjing Brain Hospital, Nanjing Medical University, Nanjing, China

**Keywords:** varicella-zoster virus, encephalitis, meningitis, myelitis, metagenomic next-generation sequencing, unimmunocompromised

## Abstract

**Background:**

Varicella-zoster virus (VZV) central nervous system infection is typically observed in immunocompromised patients, and there is a lack of studies involving large samples of non-immunocompromised individuals. In this study, we retrospectively analyzed 108 non-immunocompromised patients diagnosed with VZV central nervous system infection.

**Methods:**

This retrospective study was conducted in the Department of Neurology, Affiliated Nanjing Brain Hospital, Nanjing Medical University, China. Metagenomic next-generation sequencing (mNGS) of cerebrospinal fluid (CSF) revealed a positive result for VZV with a sequence number greater than 3, leading to a clinical diagnosis of VZV central nervous system infection. We analyzed the patients’ age, gender, clinical manifestations, blood routine, erythrocyte sedimentation rate (ESR), CSF examination, magnetic resonance imaging (MRI), electroencephalogram (EEG), and Activities of Daily Living (ADL) scale scores (Barthel Index) on the day of admission and 3 month post-discharge.

**Results:**

The study involved 108 patients, average age was 47.58 ± 2.91 years old (16 to 80), 33 were female (30.60%) and 75 were male (69.40%). Clinical manifestations were fever (63.9%), headache (88.9%), nausea (50%), vomiting (27.8%), fatigue (50%), dizziness (25.0%), herpes zoster (47.2%), chickenpox (0.9%), peripheral facial paralysis (19.4%), encephalopathy (5.6%), and myelitis (2.8%). The average white blood cell (WBC) count was 7.40 ± 0.48*10^9^/l, the average CRP was 6.58 ± 0.69 mg/L, and the average ESR was 7.79 ± 0.53 mm/h. 28.1% of patients exhibited elevated lumbar puncture pressure, the average lumbar puncture pressure was 155.41 ± 2.38 mmH_2_O; the average CSF WBC count was 196.60 ± 3.98*10^6/l, the average CSF protein was 1.35 ± 0.03 g/L, the average CSF glucose was 3.41 ± 0.03 mmoL/L, the average CSF chloride was 116.62 ± 0.15 mmoL/L, the average CSF IgG index was 0.66 ± 0.01, the average mNGS examination of VZV sequence count was 626.25 ± 5402.17. Head MRI scans revealed no new lesions; three patients’ spinal cord MRI displayed short-segment, non-transverse, and non-continuous patchy long T1 and long T2 signals in the thoracic or cervical spinal cord. On the first day of admission, 41.7% of the patients achieved ADL score of 100 points, 19.4% scored between 41 and 99 points, and 38.9% of the patients scored less than 40 points. All patients received intravenous infusions of acyclovir with low-dose corticosteroids. An outpatient review conducted 3 months after discharge indicated 98.15% of the patients recovered well without any sequelae.

**Conclusion:**

VZV encephalitis in immunocompetent individuals typically presents with mild clinical symptoms and has a favorable prognosis. VZV should be considered the common pathogen in the management of patients without immunocompeted condition with encephalitis.

## 1 Introduction

Varicella-zoster virus (VZV) is a neurotropic herpes virus belonging to the Herpesviridae family, specifically the Alphaherpesvirinae subfamily. Initial infection with VZV typically causes chickenpox in children and then remains dormant in the cranial, dorsal root, or autonomic ganglia, with the potential to reactivate and lead to conditions such as shingles. This reactivation can also lead to central nervous system infections, such as encephalitis, myelitis, or meningitis. Previous studies indicate that VZV is the second most frequent pathogen associated with infectious encephalitis, alongside other viruses such as herpes simplex virus and enterovirus (1). Patients with underlying conditions such as cancer (2, 3), organ transplantation (3), or acquired immunodeficiency syndrome (AIDS) (4), or undergoing treatments like chemotherapy or immunosuppression (5), are at a significantly increased risk for the severity of VZV encephalitis. About 50% of VZV encephalitis cases occur in immunosuppressed individuals, where viral reactivation is more common (6). This study elucidates the clinical characteristics of patients with VZV central nervous system (CNS) infection who do not exhibit immune dysfunction, conducted at a single center in southern China.

## 2 Methods

A retrospective observational study was conducted at the Department of Neurology, Affiliated Nanjing Brain Hospital, Nanjing Medical University from September 2014 to September 2023. The study included 108 patients diagnosed with central nervous system infections, those whose cerebrospinal fluid (CSF) tested positive for VZV. None of the patients had immunocompromised conditions, including those taking immunosuppressive drugs, with cancer, undergoing chemotherapy, having undergone organ transplantation, or HIV infection. The patient diagnoses adhered to the 2013 criteria established by the International Encephalitis Consortium (7). All patients tested positive with VZV DNA by mNGS in their CSF samples(VZV sequence number > 3). Participation in the study was voluntary, and all patients provided consent. Diagnosis of all patients was confirmed by at least two experienced neurologists.

Clinical data were collected from electronic medical records, which included: (1) demographic information:age and gender; (2) clinical presentation features; (3) laboratory blood results; (4) routine CSF parameters; (5) MRI and electroencephalogram (EEG) test results; and (6) Activities of Daily Living (ADL) scale scores (Barthel Index) recorded on the day of admission and 3 month post-discharge.

Statistical analysis was performed using SPSS (version 26) software (SPSS, Inc., Chicago, IL) on a MAC operating system. Quantitative variables following a normal distribution were compared using the Student’s t-test and reported as mean ± standard deviation (SD). Non-normally distributed variables were presented as median and interquartile range (IQR) and analyzed using the Mann–Whitney U test. Chi-square test was employed for comparing qualitative variables. A *p*-value <0.05 was considered statistically significant.

## 3 Results

### 3.1 AGE and SEX

The study involved 108 patients, with an average age of 47.58 ± 2.91 years. The age range of participants varied from 16 to 80 years. Among the patients, 33 were female (30.60%) and 75 were male (69.40%). The average age of female patients was 52.73 ± 5.2 years, ranging from 24 to 80 years old. Male patients had an average age of 44.44 ± 3.68 years, with an age range of 16 to 78 years. There was no significant difference in the age of onset between female and male patients (*t* = 1.27, *p* = 0.21).

### 3.2 Clinical manifestations

#### 3.2.1 Fever

A total of 69 patients (63.9%) presented with fever. Among these, 39 patients (36.1%) experienced low-grade fever (37.3–38°C), while 30 patients (27.8%) had moderate fever (38.1–39.0°C). Additionally, 39 patients (36.1%) were afebrile. The average duration of fever was 6.82 ± 0.91 days, ranging from 1 to 20 days. Specifically, the average fever duration in the low fever group was 7.46 ± 1.40 days, while in the moderate fever group it was 5.60 ± 0.87 days, statistical analysis did not reveal a significant difference between the two groups (*p* = 0.31). Age did not show a significant difference among the three groups (*p* = 0.41). It was noteworthy that all patients with moderate fever were male, a significant difference compared to the other two groups (*p* = 0.04).

#### 3.2.2 Headache, nausea and vomiting

Ninety-six patients (88.9%) presented with headache, localized in the forehead, temporal, and occipital regions. The headaches were primarily characterized by dull pain, with an average Visual Analog Scale (VAS) score of 5.34 ± 2.20, on a scale ranging from 1 to 10. Specifically, 26.9% of patients experienced mild pain (VAS 1–3), 40.4% reported moderate pain (VAS 4–6), and 32.7% had severe pain (VAS 7–10). Additionally, 54 patients (50.0%) experienced nausea, while 30 patients (27.8%) reported vomiting. The frequency of vomiting ranged from 1 to 3 times per day and persisted for a duration of 1 to 3 days.

#### 3.2.3 Fatigue and dizziness

Ninety patients (83.3%) reported experiencing fatigue; however, most patients’ daily living activities remained unaffected, with only one-third of patients experiencing an impact on their work ability. Additionally, 27 patients (25.0%) reported experiencing dizziness.

#### 3.2.4 Herpes zoster, chicken pox and peripheral facial paralysis

Fifty-one patients (47.2%) were diagnosed with herpes zoster. The average age of patients with herpes was 52.59 ± 7.28 years, ranging 21 to 80 years old. In contrast, the average age of patients without herpes was 41.95 ± 5.02 years, ranging 16 to 62 years old, no significant difference was observed between the two groups (*t* = −1.78, *p* = 0.087). Thirty patients exhibited herpes in the distribution area of the trigeminal nerve, with all affected nerves being branches of the first division of the trigeminal nerve. Additionally, 21 patients presented with herpes in the spinal nerve regions: 16 patients had lesions located on the chest and back, corresponding to the thoracic spinal nerve distribution, while 5 patients had lesions on the thigh or calf, corresponding to the lumbar spinal nerve distribution. Only one 16-year-old male patient presented with chickenpox. Twenty-one patients (19.4%) experienced peripheral facial paralysis, 16patients had herpes in the trigeminal nerve region and 5 patients had no herpes. The risk of facial paralysis in patients with herpes affecting the trigeminal nerve area is 50.0%, compared to a 10.5% risk in patients without herpes. Notably, no patients with herpes in the spinal nerve area develop facial paralysis. The risk of facial paralysis in patients with herpes in the trigeminal nerve area is significantly higher than in those with herpes in the spinal nerve or in patients without herpes (*p* = 0.013).

#### 3.2.5 Encephalopathy

Six patients (5.6%) exhibited encephalopathy, included impairments in recent memory, orientation, and comprehension. The average Mini-Mental State Examination (MMSE) score among these patients was 19.00 ± 0.77 points. This encephalopathy condition persisted for a duration of 3 to 9 days, with an average duration of 5.83 ± 0.91 days. Patients experiencing encephalopathy were typically accompanied by psychiatric symptoms, which manifested as emotional instability, increased restlessness, and the absence of hallucinations or abnormal behaviors.

#### 3.2.6 Limb weakness and difficulty urinating

Three patients (2.8%) presented with myelitis, all of whom exhibited short-segment, non-transverse myelitis. All three patients demonstrated weakness in both lower limbs; one patient had a muscle strength grade of 2 in both lower limbs, while the other two patients had a muscle strength grade of 4. Two patients were unable to relieve their bowels, with one requiring a urinary catheter for 1 month and the other for 2 weeks.

**Figure 1:**
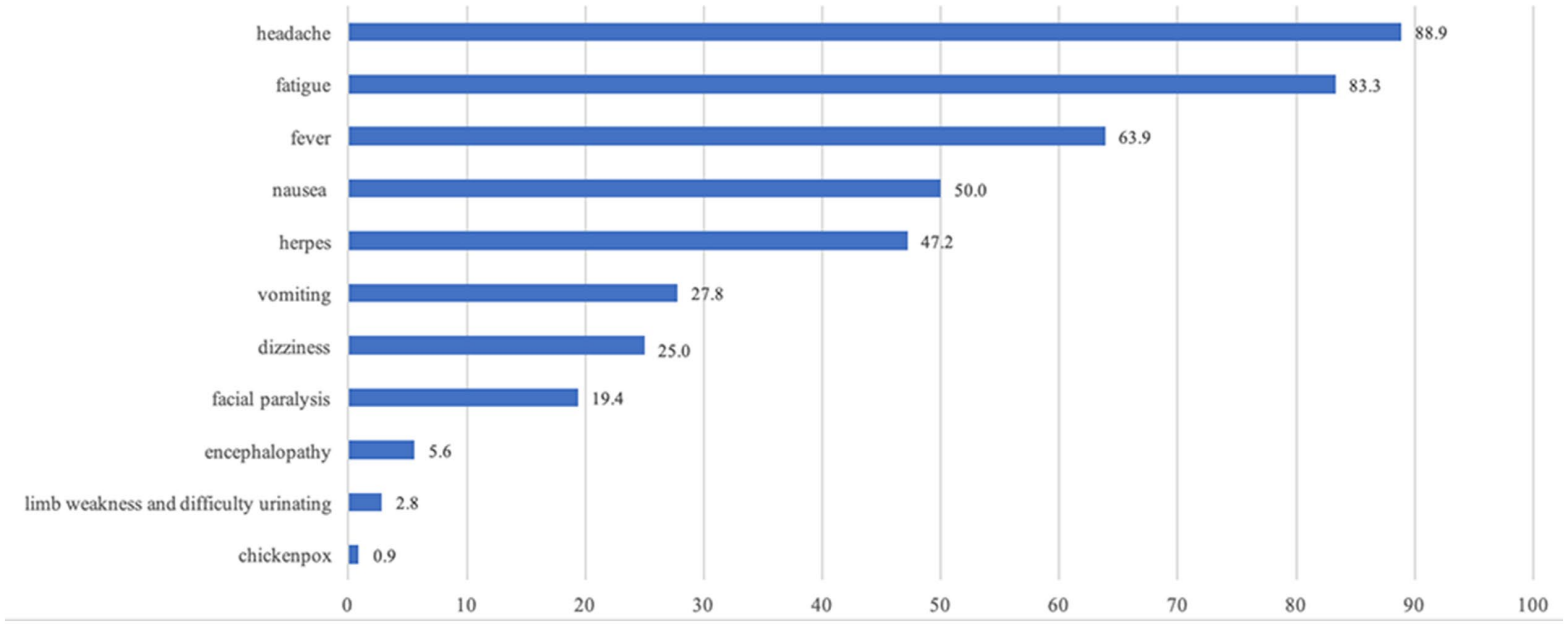
Clinical manifestations of all patients (%).

**Figure 2:**
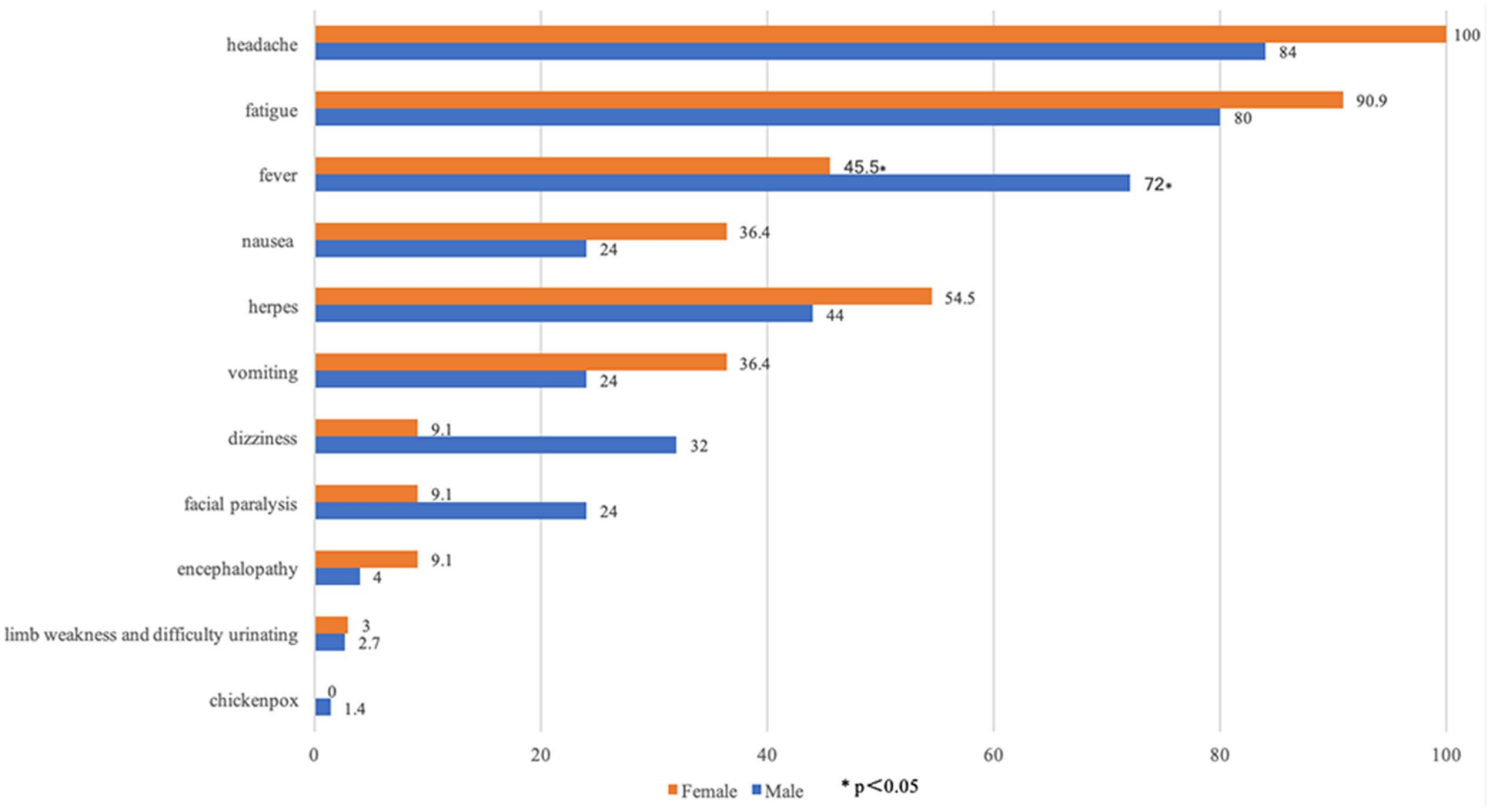
Clinical manifestations grouped by gender (%).

**Figure 3:**
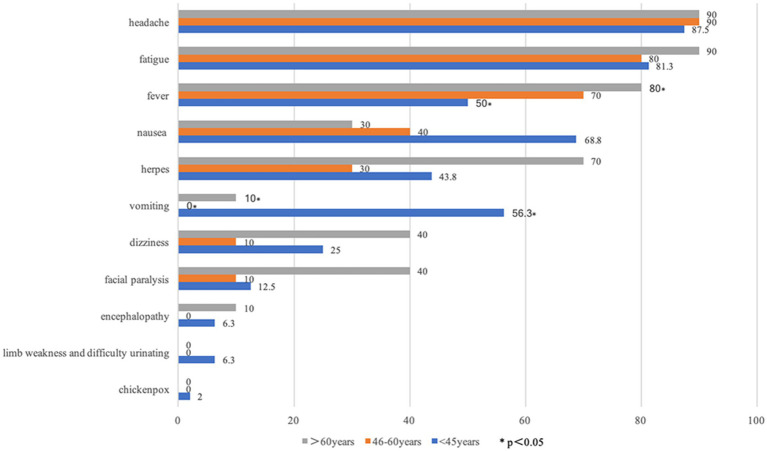
Clinical manifestations grouped by years (%).

The clinical manifestations observed in our patients show in Figure 1. We categorized the patients by gender (Figure 2) and age (Figure 3), revealing that the fever rate among male patients (72.0%, 54/75) was significantly higher than that of female patients (45.5%, 15/33) (*p* = 0.008). Additionally, the fever rate in young patients (50%, 24/48) was significantly lower than that in middle-aged patients (70.0%, 21/30) and elderly patients (80.0%, 24/30) (*p* = 0.020). The incidence of vomiting in young patients (56.3%, 27/48) was significantly higher than in middle-aged patients (0%) and elderly patients (10.0%, 3/30) (*p* = 0.003).

### 3.3 Laboratory examination

#### 3.3.1 Routine blood tests, serum C-reactive protein and erythrocyte sedimentation rate

**Figure 4:**
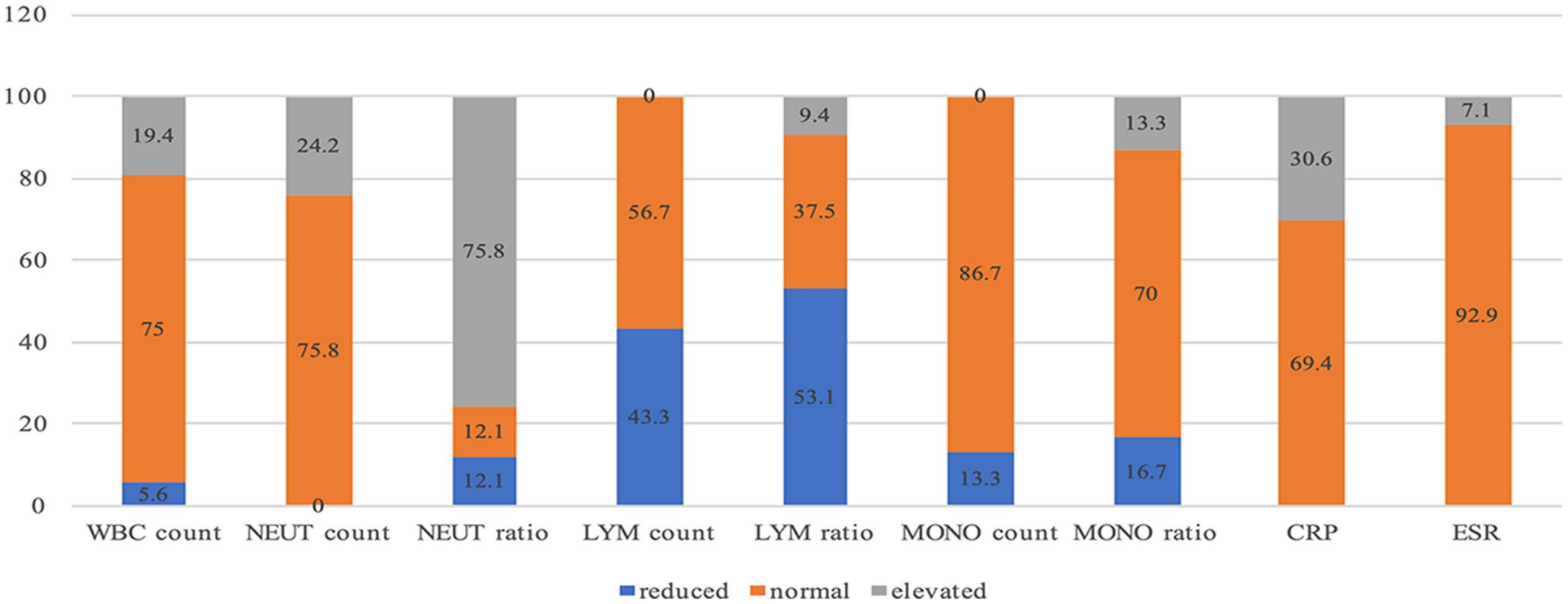
The proportion of Elevated, Normal, and Reduced Routine Blood Tests, ESR, and CRP (%).

The ESR in female patients was 13.50 ± 1.26 mm/H, significantly higher than that of male patients, which was 5.50 ± 0.42 mm/H (*p* = 0.015). The average blood CRP level in elderly patients was 5.74 ± 0.69 mg/L, notably higher than that of middle-aged patients at 4.21 ± 0.99 mg/L and young patients at 0.65 ± 0.12 mg/L (*p* = 0.033). The proportion of elevated WBC counts in patients with spinal nerve regional herpes (42.9%) was significantly higher than in patients with trigeminal nerve herpes (0%) and those without herpes (21.1%) (*p* = 0.049). Additionally, the proportion of decreased WBC counts in patients with trigeminal nerve herpes (20%) was significantly higher than in patients with herpes and no herpes in the spinal nerve area (*p* = 0.049). Neutrophil counts were elevated in 57.1% (12/21) of patients with herpes in the spinal nerve region, significantly higher than in patients with trigeminal nerve herpes (0%) and those without herpes (23.5%) (*p* = 0.030; Table 1; Figure 4).

**Table 1:** Average, minimum, and maximum values of blood routine, CRP, and ESR.

	Average	Minimum	Maximum
WBC (*10^9/l)	7.40 ± 0.48	3.35	11.83
NEUT count (*10^9/l)	5.26 ± 0.21	1.61	9.07
NEUT ratio (%)	71.33 ± 1.99	46.36	89.94
LYM count (*10^9/l)	1.38 ± 0.06	0.40	3.14
LYM ratio (%)	20.78 ± 1.85	6.22	43.12
MONOcount (*10^9/l)	0.44 ± 0.02	0.08	0.85
MONO ratio(%)	6.93 ± 0.84	1.21	12.78
CRP (mg/l)	6.58 ± 0.69	0.50	54.06
ESR (mm/h)	7.79 ± 0.53	2.00	24.00

WBC, White blood cells; NEUT, neutrophils; LYM, lymphocytes; MONO, monocytes; CRP, C-reactive protein; ESR, erythrocyte sedimentation.

### 3.4 Cerebrospinal fluid examination

**Figure 5:**
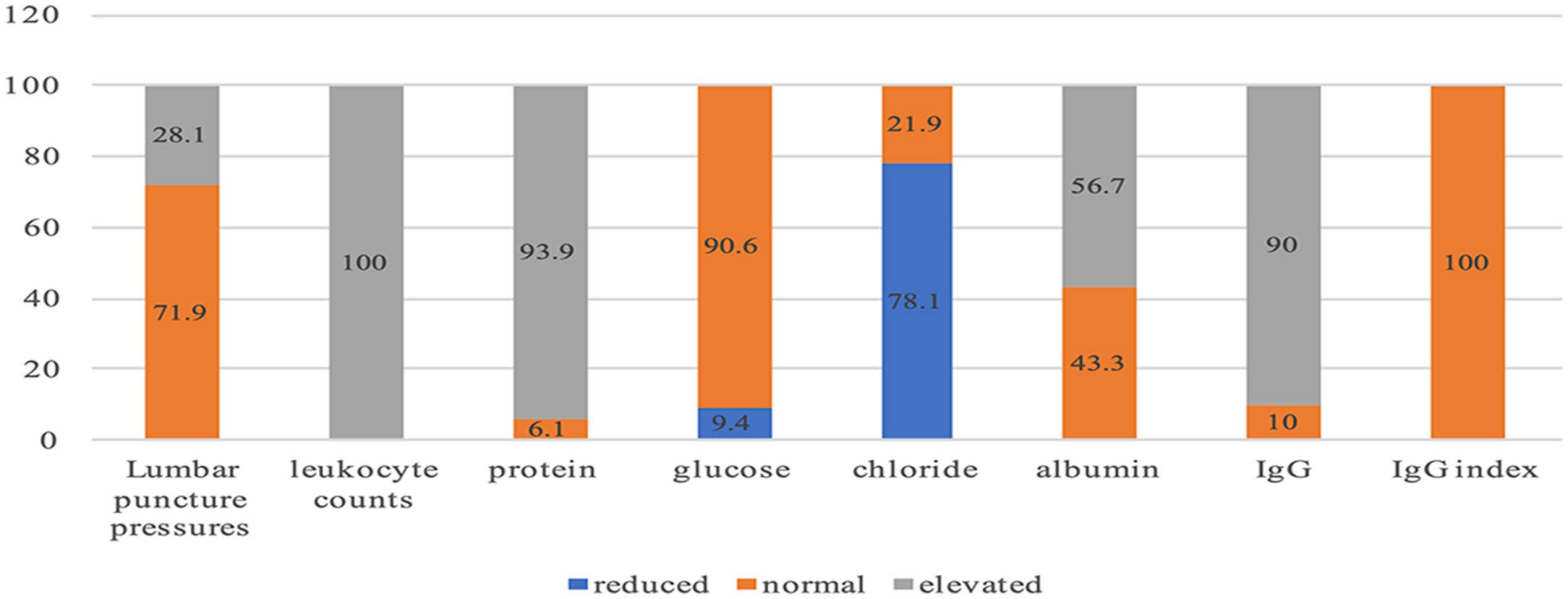
The proportion of Elevated, Normal and Reduced CSF examination (%). lumbar puncture pressure > 180 mmH20 is considered elevated, 80–180 mmH20 is considered normal; leukocyte counts >6*10^6^ /L is considered elevated; protein >0.4 g/L is considered elevated, 0.2–0.44 g/L is considered normal; glucose<2.5 mmoL/L is considered reduced, 2.5–4.4mmo/l is considered normal; chloride <120 mmoL/L is considered reduced, 120-130 mmo/l is considered normal; albumin >350 mg/L is considered elevated, <350 mg/L is considered normal; IgG >34 mg/L is considered elevated, <34 mg/L is considered normal; IgG index = (IgG CSF/IgG serum)/(albumin CSF/albumin serum), IgG index >0.85 is considered elevated, <0.85 is considered normal. CSF-IgG detection in our center utilizes a rate scattering immune ratio. “34 mg/L “is the cut-off value given by our center’s laboratory. “IgG >34 mg/L” represents an increase in CSF IgG, which may occur during central nervous system infections or inflammations. “IgG l < 34 mg/L “is considered to be within the normal range.

Notably, 28.1% of patients exhibited elevated lumbar puncture pressure, and all of them were male (*p* = 0.018). The average lumbar puncture pressure in male patients was 168.82 ± 9.32 mmHg, it was significant higher than female patients’ 125.90 ± 7.86 mmHg (*p* = 0.004). The average CSF leukocyte counts in the young group (<45 years old), the middle-aged group(45–60 years old) and the elderly group(45–60 years old) respectively was 273.44 ± 22.78 × 10^6/l, 169.33 ± 11.87 × 10^6/l and 98.20 ± 11.86 × 10^6/l; the elderly group’s CSF leukocyte count was significantly lower than that of the other two groups (*p* = 0.009). The average CSF glucose level in the young group was 2.92 ± 0.06 mmoL/L, significantly lower than that in the middle-aged group (4.01 ± 0.34 mmoL/L) and the elderly group (3.78 ± 0.38 mmoL/L) (*p* = 0.012). The average CSF chloride levels in the young group was 118.89 ± 0.71 mmoL/L, significantly higher than those in the middle-aged group (113.90 ± 1.89 mmoL/L) and the elderly group (114.78 ± 1.41 mmoL/L) (*p* = 0.008). The average CSF IgG index in female patients was 0.73 ± 0.06, significantly higher than that of male patients’ 0.52 ± 0.04 (*p* = 0.048). The metagenomic next-generation sequencing (mNGS) examination of CSF showed the average VZV sequence count was 626.25 ± 5402.17, ranging from 3 to 24,580 (Table 2; Figure 5).

**Table 2:** Average, minimum, and maximum values of CSF examination.

	Average	Minimum	Maximum
Lumbar puncture pressures (mmH2O)	155.41 ± 2.38	70	350
Leukocyte counts (*10^9/l)	196.60 ± 3.98	8	865
Mononuclear cells (%)	96.06 ± 0.11	89	99
Polynuclear cells (%)	3.97 ± 0.11	1	11
Protein (g/l)	1.35 ± 0.03	0.26	5.68
Glucose (mmol/l)	3.41 ± 0.03	2.04	6.01
Chloride (mmol/l)	116.62 ± 0.15	107	124
Albumin (mg/l)	715.77 ± 20.02	150	2,539
IgG (mg/l)	112.47 ± 3.38	30.6	493
IgG index	0.66 ± 0.01	0.43	1
mNGS VZV sequence count	626.25 ± 5402.17	3	245,830

### 3.5 MRI and electroencephalogram tests

All patients underwent head MRI scans, which revealed no new lesions; however, some patients exhibited signs of old lacunar infarctions. Three patients also underwent spinal cord MRI. Of these, one patient displayed short-segment, non-transverse, and non-continuous patchy long T1 and long T2 signals in the thoracic spinal cord, while the other two patients presented with non-transverse myelitis in the cervical spinal cord. The EEG results showed that 61.1% of patients were within the normal range, while 38.9% exhibited mildly abnormal EEG findings, characterized by increased theta waves and an irregular alpha rhythm.

### 3.6 Activity of daily living scale

On the first day of admission, we assessed the patient’s ADL score, which was evaluated using the Barthel Index. 41.7% of the patients achieved a perfect ADL score of 100 points; while 19.4% scored between 41 and 99 points, indicating a need for slight assistance in daily activities; 38.9% of the patients scored less than 40 points, signifying a requirement for substantial assistance in their daily lives.

### 3.7 Treatment and prognosis

All patients received intravenous infusions of antiviral at a dosage of 0.5 g every 8 h for a duration of 14–21 consecutive days, in conjunction with low-dose corticosteroids (either methylprednisolone at 80 mg/day or dexamethasone at 10 mg/day) for 7 days. Three patients presented with myelitis received antiviral treatment for an extended period of 35 to 42 days, along with corticosteroid therapy for 21 days. An outpatient review conducted 3 months after discharge indicated that 98.15% of the patients recovered well without any sequelae. One patient experienced mild weakness in both lower limbs (muscle strength level V-) and poor urinary control. Additionally, one patient died due to unrelated causes.

### 3.8 Data analysis

#### 3.8.1 Comparative analysis

Based on the main clinical manifestations, patients were categorized into three groups: meningitis, encephalitis, and myelitis. We compared the clinical manifestations, blood routine results, and CSF examination findings across three groups of patients (Table 3).

**Table 3:** General information, clinical manifestation, blood routine examination and CSF examination in meningitis, encephalitis, and myelitis group.

		Meningitis group *n* = 96	Encephalitis group *n* = 9	Myelitis group *n* = 3	*p* value
General information	Age (years)	45.94 ± 3.00	56.33 ± 6.19	43.28 ± 8.95	0.63
	Male *n* (%)	66 (68.8)	6 (66.7)	2 (66.7)	0.80
	Female *n* (%)	30 (31.3)	3 (33.3)	1 (33.3)	0.80
Clinical manifestation	Fever *n* (%)	60 (62.5)	6 (66.7)	1 (33.3)	0.74
	Headache *n* (%)*	**93 (96.9)**	**3 (33.3)**	**0**	**0.00**
	VAS score	5.33 ± 0.39	4.05 ± 0.12	–	0.45
	Fatigue *n* (%)	81 (84.4)	6 (66.7)	3 (100)	0.66
	Nauses *n* (%)	51 (53.1)	3 (33.3)	0	0.48
	Vomiting *n* (%)	27(28.1)	3 (33.3)	0	0.82
	Herpes *n* (%)	42(43.8)	6 (66.7)	2 (66.7)	0.42
	Trigeminal herpes *n* (%)*	**30 (31.3)**	**0**	**0**	**0.05**
	Spinal herpes *n* (%)*	**12 (12.5)**	**6 (66.7)**	**2 (66.7)**	**0.05**
	Dizziness *n* (%)	27 (28.1)	0	0	0.47
	Facial paralysis *n* (%)	21 (21.9)	0	0	0.58
	Encephalopathy *n* (%)*	**0**	**6 (66.7)**	**0**	**0.01**
	Chickenpox *n* (%)	0	1 (11.1)	0	0.12
	Positive meningeal irritation sign *n* (%)	42 (43.8)	3 (33.3)	0	0.67
	ADL score on the day of admission	90.00 ± 26.33	45.00 ± 14.65	33.33 ± 9.87	0.36
	100 points *n* (%)	39 (40.6)	3 (33.3)	0	0.67
	99–41 points *n* (%)	21 (21.9)	0	0	0.67
	<40 points *n* (%)	36 (37.5)	6 (66.7)	3 (100)	0.67
Blood routine examination	WBC count (*10^9/l)*	**6.92 ± 0.38**	**11.04 ± 0.34**	**9.15 ± 0.27**	**0.01**
	>10 *n* (%)*	**12 (12.5)**	**9 (100)**	**0**	**0.01**
	4–10 *n* (%)*	**78 (81.3)**	**0**	**3 (100)**	**0.01**
	<4 *n* (%)*	**6 (6.3)**	**0**	**0**	**0.01**
	NEUT count (*10^9/l)	5.05 ± 0.38	7.03 ± 0.55	6.93 ± 0.64	0.21
	>6.13 *n* (%)*	**13 (13.5)**	**6 (66.7)**	**3 (100)**	**0.00**
	1.56–6.13 *n* (%)*	**83 (86.5)**	**3 (33.3)**	**0**	**0.00**
	<1.56 *n* (%)	0	0	0	-
	LYM count (*10^9/l)	1.34 ± 0.10	1.93 ± 1.21	1.28 ± 0.21	0.42
	>3.74 *n* (%)	0	0	0	0.67
	1.18–3.74 *n* (%)	53 (55.2)	3 (50.0)	3 (100)	0.67
	<1.18 *n* (%)	43 (44.8)	3 (50.0)	0	0.67
	MONO count (*10^9/l)	0.45 ± 0.03	0.49 ± 0.37	0.30 ± 0.05	0.76
	>0.82 *n* (%)	0	0	0	0.27
	0.24–0.82 *n* (%)	85 (88.5)	3 (50.0)	3 (100)	0.27
	<0.24 *n* (%)	11 (11.5)	3 (50.0)	0	0.27
	NEUT ratio	71.80 ± 3.53	78.97 ± 8.92	76.59 ± 5.85	0.32
	>65% *n* (%)	66 (75.9)	6 (66.7)	3 (100)	0.74
	50–65% *n* (%)	9 (10.3)	3 (33.3)	0	0.74
	<50% *n* (%)	12 (13.8)	0	0	0.74
	LYM ratio	19.90 ± 3.63	19.10 ± 7.07	14.40 ± 0.53	0.76
	<20% *n* (%)	45 (51.7)	3 (50.0)	3 (100)	0.88
	20–40% *n* (%)	33 (37.9)	3 (50.0)	0	0.88
	>40% *n* (%)	9 (10.3)	0	0	0.88
	MONO ratio	6.70 ± 0.52	4.75 ± 3.35	3.40 ± 2.06	0.36
	<4% *n* (%)	9(11.1)	3 (50.0)	3 (100)	0.12
	4–10% *n* (%)	60 (74.1)	3 (50.0)	0	0.12
	>10% *n* (%)	12 (14.8)	0	0	0.12
	CRP (mg/l)*	**1.08 ± 1.32**	**33.60 ± 16.66**	**0.51 ± 0.04**	**0.00**
	0–8	69 (71.9)	3 (33.3)	3 (100)	0.31
	>8	27 (28.1)	6 (66.7)	0	0.31
	ESR (mm/h)	8.07 ± 1.08	–	–	0.53
	0–15/20	36 (92.3)	3 (100)	–	0.77
	>15/20	3 (7.7)	–	–	0.77
CSF examination	Pressure (mmH20)*	**159.86 ± 2.45**	**79.00 ± 3.79**	**168.00 ± 25.50**	**0.05**
	80–180 *n* (%)	72 (75.0)	9 (100)	0	0.14
	>180 *n* (%)	24 (25.0)	0	3 (100)	0.14
	Leukocyte counts (*10^6/l)	121 ± 69.33	40.33 ± 18.85	278.45 ± 50.78	0.38
	Mononuclear cells (%)*	**96.50 ± 0.11**	**91.00 ± 1.53**	**98.67 ± 0.56**	**0.01**
	Polynuclear cells (%)*	**3.53 ± 0.10**	**9.00 ± 1.53**	**1.33 ± 0.56**	**0.01**
	Protein (g/l)	1.16 ± 0.42	0.69 ± 0.17	0.73 ± 0.15	0.37
	0.2–0.4	3 (3.1)	3 (33.3)	0	0.12
	>0.4	93 (96.8)	6 (66.7)	3 (100)	0.12
	Glucose (mmol/l)	3.25 ± 0.34	3.16 ± 0.33	2.89 ± 0.25	0.91
	<2.5	10 (10.4)	0	0	0.79
	2.5–4.4	86 (89.6)	9 (100)	3 (100)	0.79
	Chloride (mmol/l)	116.73 ± 0.80	118.73 ± 1.66	116.20 ± 0.38	0.06
	<120	75 (78.1)	6 (66.7)	3 (100)	0.77
	120–130	21 (21.9)	3 (33.3)	0	0.77
	albumin(mg/l)	453.00 ± 288.5	345.00 ± 97.69	1223.00 ± 448.85	0.34
	0–350	44 (45.8)	3 (33.3)	0	0.62
	>350	52 (54.2)	6 (66.7)	3(100)	0.62
	IgG (mg/l)	86.75 ± 40.36	59.07 ± 13.76	236.00 ± 20.78	0.29
	0–34	7 (7.3)	3 (33.3)	0	0.35
	>34	89 (92.7)	6 (66.7)	3 (100)	0.35
	IgG index	0.64 ± 0.05	0.58 ± 0.07	0.85 ± 0.08	0.17
	mNGS VZV sequence count*	**465.5 ± 1723.67**	**30843.33 ± 8188.73**	**16577.37 ± 2097.87**	**0.03**

Items with significant differences are highlighted in bold.*p < 0.05. WBC, White blood cells; NEUT, neutrophils; LYM, lymphocytes; MONO, monocytes; CRP, C-reactive protein; ESR, erythrocyte sedimentation; VAS, Visual Analog Scale; ADL, Activities of Daily Living.

The proportions of headaches and herpes zoster in the trigeminal nerve area among patients in the meningitis group were significantly higher than those in the other two groups (*p* < 0.05). The proportions of herpes zoster in the spinal nerve area were significantly greater in the encephalitis and myelitis groups compared to the meningitis group (*p* < 0.05). The incidence of encephalopathy was significantly higher in the encephalitis group than in the other two groups (*p* < 0.05). WBC count, neutrophil count, and CRP levels in the encephalitis group were significantly elevated compared to both the meningitis and myelitis groups (*p* < 0.05). The lumbar puncture pressure in the encephalitis group was significantly lower than that in the other two groups (*p* < 0.05). Finally, the CSF-VZV sequence count in patients in encephalitis group was significantly higher than that observed in both the meningitis and myelitis groups (*p* < 0.05).

## 4 Discussion

The incidence of presumed infectious encephalitis is estimated to range between 1.5 and 7 cases per 100,000 people annually, excluding epidemics (1). The most common etiological agents include herpesviruses 1 and 2, enteroviruses, arboviruses, influenza virus, cytomegalovirus, Epstein–Barr virus, human herpesvirus 6, and the re-emergent measles virus (1). According to the Consensus Statement of the International Encephalitis Consortium published in 2013, PCR testing methods are recommended to elucidate the etiology (7). A review of encephalitis literature from 2000 to 2015 revealed that between 21 and 72% of cases remain undiagnosed (1). The increasing adoption of metagenomic next-generation sequencing (mNGS) has led to a growing number of confirmed etiological diagnoses of viral encephalitis, significantly enhancing clinical diagnostic capabilities (8). However, VZV may yield negative results early in the infection (9), so repeat testing with a separate CSF specimen is advised 3–7 days later when there is a strong clinical suspicion of VZV encephalitis (10). It’s important to note that detection results can be complicated by the presence of other viruses, such as human herpesvirus 6 or cytomegalovirus, in the CSF of immunologically competent patients (11).

After primary infection, VZV becomes latent in ganglionic neurons along the entire neuraxis. With a decline in VZV-specific cell-mediated immunity, VZV reactivates from ganglia and travels anterograde to the skin to cause zoster, the trigeminal, cervical, and thoracic sensory nerves are the most common nerves involved in VZV reactivation. In our study, 47.2% (51/108) patients had herpes zoster, among these, 30 patients exhibited herpes lesions confined to the first branch of the trigeminal nerve. Additionally, 16 patients had lesions within the thoracic nerve distribution area, while 5 patients had lesions in the lumbar nerve distribution area. VZV can also travel retrograde to produce meningoencephalitis, myelitis, and stroke (12). VZV encephalitis in adults is primarily caused by viral reactivation, with central nervous system manifestations potentially appearing before, during, or after the onset of dermatomal zoster lesions. In our study, approximately one-third of the patients experienced herpes before encephalitis or meningitis symptoms. Notably, VZV encephalitis or meningitis can occur without the typical skin findings, which has been reported in up to one-third of cases (13), which may suggest that a direct viral invasion of the leptomeninges from the spinal ganglia. In our group, 52.8% (57/108) of individuals did not have herpes zoster. 42.6% (29/68) of participants in Maille’s four-year prospective cohort study conducted in France from 2016 to 2019 also did not have herpes zoster (14), which is consistent with our findings. 22% of patients in Adrien Milrose’s study were reported without herpes zoster (15). In Gha-Hyun Lee’s study (16), 56.1%(23/41)did not have herpes zoster. When these patients occur without rash, VZV-induced disease can be diagnosed by detection of VZV DNA or anti-VZV antibody in CSF.

In our study, none of patients was immunosuppressed or admitted to intensive care unit (ICU), 30.6% of the patients were female, 69.4% were male, the average age was 47.58 years old (range 16–80 years old). A French study (15), all patients were hospitalized in ICU and 78% of them were immunosuppressed, 47% of the patients were female and 53% were male, the age ranged from 36 to 66 years old, with an average of 53 years old, the age is slightly higher than patients in our study, while the gender distribution remains similar. The findings of the other two studies align closely with our observations regarding age and gender (16, 17). No seasonal patterns were observed in our study, is consistent with the study of Gha-Hyun (16).

In our group, 36.1% (39/108) of patients exhibited no fever, which aligns with the finding of 30.9% (21/68) of patients in Maille’s study who also exhibited no fever (14). The most common neurologic symptoms of VZV infection are meningoencephalitis, cerebellitis, stroke, myelopathy, and retinitis (7, 18). In our study, 88.9% (98/108) of patients presented with meningitis, 8.3% (9/108) with encephalitis, and 2.8% (3/108) with myelitis. Among the patients in Chile, a single-center retrospective study revealed that clinical presentation was meningitis in 68.75% (11/16) of patients and encephalitis in 31.25% (5/16) of patients (17). However, in Gha-Hyun Lee’s study, 83.3% (35/42) patients presented with meningitis and 16.7% (7/42) patients presented with encephalitis (16).

CSF pleocytosis was observed in all cases within our group, consistent with previous studies (17, 19). In Gha-Hyun Lee’s study, the average CSF WBC count in patients with VZV encephalitis was reported to be 301 × 10^6 cells/L (16), which was higher than the 196.60 × 10^6 cells/L observed in our study. Due to technical limitations, mNGS cannot entirely eliminate the possibility of false negative and false positive results. To reduce the risk of false positives, we defined a threshold of mNGS-VZV >3 as indicative of a positive result. In our study, the average mNGS-VZV sequence count was 626.25 ± 5402.17, with a range from 3 to 24,580. Currently, there are no other research data reports available for comparison. Further investigation is necessary to ascertain whether this count accurately reflects the disease characteristics of VZV encephalitis. Combining CSF-mNGS and CSF VZV-IgM may reduce the incidence of false positives or false negative. At our center, patients suspected of having a CNS infection typically undergo a lumbar puncture for CSF examination within 1 to 3 days of admission. CSF specimens were collected for WBC count, protein, chloride, glucose, albumin, and immunoglobulin G (IgG). For patients with CSF-WBC >6 × 10^9/L, further identification of the etiology was pursued. Some patients consented to undergo CSF-mNGS testing. For those with negative results, it was recommended to complete the detection of IgG/IgM pathogens in both blood and CSF. Typically, the result of CSF-mNGS was available within 1 to 2 days. When there was a need to continue IgG/IgM detection of pathogens in blood and CSF, patients hospitalized for 3 to 7 days, and many experienced symptom improvement, which led to reluctance in proceeding with IgG/IgM detection of pathogens in blood and CSF. Consequently, extremely few patients underwent IgG/IgM detection of blood and CSF pathogens at our center. Due to the limitations of our testing technology, our center does not routinely detect oligoclonal bands. Therefore, in our research, there were no VZV-IgG/IgM or oligoclonal bands. Notably, our study found no lesions on brain MRI in patients with meningitis or encephalitis; only three patients, whose primary manifestation was myelitis, exhibited relevant lesions on spinal cord MRI. In contrast, Gha-Hyun Lee’s study (16), noted that 4.8% (2/42) of patients had abnormal imaging: one patient displayed multifocal nodular enhancement on T1-weighted enhanced images, while another exhibited high signal intensity with hemorrhagic transformation in the left inferior frontal lobe and temporal lobe. The lesions associated with VZV encephalitis were located in multiple areas, predominantly affecting the parietal lobe, followed by the frontal and temporal lobes. These lesions may present as ischemic stroke, intracerebral hemorrhage, or venous sinus thrombosis due to vasculopathy (20–22), with ischemic stroke being the most common cerebrovascular complication after VZV encephalitis (22). Our findings indicate that viral load is associated with the presence of skin herpes and encephalopathy, revealing that patients exhibiting skin herpes and encephalopathy demonstrate higher viral loads. Conversely, Anna Persson’s study (19) did not find a correlation between viral load and patients’ symptoms.

There is universal agreement among domestic and international encephalitis guidelines that intravenous acyclovir should be initiated when viral encephalitis or meningitis is suspected. The recommended dosage is 10–15 mg/kg of intravenous acyclovir every 8 h, administered continuously for 10 to 14 days. However, if progressive neurologic symptoms or radiographic progression occurs during treatment, consideration should be given to extending the duration of treatment to 4 to 6 weeks (10). The routine use of corticosteroids in the treatment of infectious encephalitis remains a topic of controversy. Some studies indicate that the administration of empiric corticosteroids does not improve outcomes in cases of infectious encephalitis (23). At our clinical center, corticosteroids are not routinely administered for VZV encephalitis; however, low-dose corticosteroids are often utilized when cerebrospinal fluid protein levels exceed 1.0 g/L.

Approximately 27% of patients with HSV encephalitis may develop secondary autoimmune encephalitis within 3 months, typically occurring within 2 months after antiviral treatment (18). However, there have been no reported cases of secondary autoimmune encephalitis following VZV infection. In our study, none of the patients developed secondary autoimmune encephalitis. 90% of patients have elevated CSF IgG, supporting central nervous system infections, however, none of the patients in our study exhibited an increased IgG index, which supports the low risk of developing autoimmune encephalitis secondary to VZV encephalitis. Notably, 98.15% (106/108) of the patients recovered well and exhibited no sequelae during the outpatient follow-up conducted 3 months after discharge; only 1 patient experienced difficulty urinating and demonstrated weak lower limb strength (grade IV). The symptoms basically returned to normal at 1 year after discharge. However, 68% (34/50) had neurological symptoms 1 month after acute disease and 50% (25/50) had neurological complications 3 months after discharge in Anna Persson’s study (19), however, specific details regarding these complications were not provided. In Maille’s study, 12.31% (8/65) of patients with VZV encephalitis succumbed to the illness (14). In contrast, our study reported a mortality rate of only 0.93% (1/108), while a study conducted in Chile reported no fatalities (17).

Our study found several noteworthy phenomena: (1) Male and elderly patients were more likely to present with fever; (2) Young patients were more prone to experiencing vomiting; (3) Patients with spinal nerve regional herpes tended to exhibit higher WBC and neutrophil counts, whereas those with trigeminal nerve herpes were more likely to show lower WBC counts; (4) The CSF pressure in male patients was significantly higher than that in females; (5) Young patients demonstrated a significantly higher CSF WBC count compared to middle-aged and elderly patients; (6) The CSF-VZV sequence count in the encephalitis group was significantly higher than that observed in both the meningitis and myelitis groups (*p* < 0.05). These phenomena may reflect the characteristics of VZV central nervous system infection, but it need to be verified by more patients. It should be noted that, mNGS cannot entirely eliminate the possibility of false negative and false positive results. The results reported by mNGS need to be comprehensively judged based on clinical patient conditions and other test results, and should not be used as direct diagnostic basis. In the future, we will conduct more in-depth research on the possible false positives or false negatives of mNGS.

## 5 Conclusion

VZV encephalitis in immunocompetent individuals typically presents with mild clinical symptoms and has a favorable prognosis. Our findings confirm that varicella-zoster virus (VZV) should be considered the common pathogen in the management of patients with encephalitis. In clinical practice, when encountering patients exhibiting symptoms such as fever, headache, herpes, encephalopathy, and myelitis, it is essential to consider the possibility of VZV-related central nervous system damage. The application of novel techniques, such as cerebrospinal fluid metagenomic next-generation sequencing (CSF-mNGS), will enhance diagnostic accuracy. Following a specific course of antiviral treatment, nearly all patients recover well without any sequelae. For patients suspected of VZV central nervous system infections, antibiotic treatment should be avoided.

## Data Availability

The raw data supporting the conclusions of this article will be made available by the authors, without undue reservation.
